# Coronary Artery Spasm in Patients with Type 2 Diabetes Mellitus

**DOI:** 10.3390/life16020354

**Published:** 2026-02-19

**Authors:** Theodor Iulian Matei, Minerva Codruta Badescu, Alexandru Dan Costache, Ionuț Tudorancea, Ionela Lăcrămioara Șerban, Sandu Cucută, Bianca-Ana Dmour, Raluca Daria Mitea, Radu-George Ciorap, Ciprian Rezus, Irina Iuliana Costache-Enache

**Affiliations:** 1 “Grigore T. Popa” University of Medicine and Pharmacy, 700115 Iasi, Romania; matei.theodor-iulian@d.umfiasi.ro (T.I.M.); dan-alexandru.costache@umfiasi.ro (A.D.C.); ionut.tudorancea@umfiasi.ro (I.T.); ionela.serban@umfiasi.ro (I.L.Ș.); cucuta.sandu@d.umfiasi.ro (S.C.); bianca-ana.gherasim-dmour@d.umfiasi.ro (B.-A.D.); radu.ciorap@umfiasi.ro (R.-G.C.); ciprian.rezus@umfiasi.ro (C.R.); irina.costache@umfiasi.ro (I.I.C.-E.); 2 Cardiology Clinic, “St. Spiridon” County Emergency Clinical Hospital, 700111 Iasi, Romania; 3 III Internal Medicine Clinic, “St. Spiridon” County Emergency Clinical Hospital, 700111 Iasi, Romania; 4 Clinical Rehabilitation Hospital, 700661 Iasi, Romania; 5 “Lucian Blaga” University, Faculty of Medicine, 550024 Sibiu, Romania; daria.mitea@ulbsibiu.ro

**Keywords:** epicardial spasm, microvascular spasm, coronary spasm, vasospastic angina, microvascular angina, diabetes mellitus, acetylcholine, ergonovine

## Abstract

Cardiovascular disease and diabetes mellitus have now reached pandemic proportions, and their association has become very common. Some patients with coronary artery disease and diabetes remain symptomatic, with chest pain present despite the implementation of evidence-based medical treatment and maximal therapy. A hypothesis increasingly confirmed in clinical practice is that epicardial or microvascular spasm, or both, are frequently responsible for the lack of symptom control or for the occurrence of major adverse cardiovascular events. Our review provides the most up-to-date and in-depth analysis of the available literature to provide solid knowledge about coronary spasm in diabetes patients. We have deepened the substrate of coronary spasm in relation to the multiple and complex structural and functional abnormalities produced by chronic hyperglycemia, with the ultimate goal of allowing optimization of treatment and improving patient outcomes.

## 1. Introduction

Coronary artery disease (CAD) has long ceased to be just a feature of advanced atherosclerosis affecting older individuals. Although myocardial ischemia caused by the complications of atherosclerotic plaques still dominates the etiology of CAD in the general population, new entities have been described in relation to signs and symptoms of ischemia in the absence of detectable atherosclerotic coronary obstruction in traditional coronary angiography. Angina with non-obstructive coronary arteries (ANOCA), ischemia with non-obstructive coronary arteries (INOCA), and myocardial infarction with non-obstructive coronary arteries (MINOCA) are now recognized as an important part of the CAD spectrum [1,2]. Their substrate is complex and includes coronary vasospasm, microvascular dysfunction, and abnormalities in blood flow regulation [3].

Patients with advanced atherosclerosis typically present with multiple traditional cardiovascular risk factors, in various combinations—older age, male gender, hypertension, dyslipidemia, diabetes mellitus, obesity, physical inactivity, smoking, and family history of premature heart disease [4]. The profile of patients with ANOCA/INOCA/MINOCA is different from the classic profile of patients with atherosclerotic CAD. These patients are mostly women, are younger, and have a lower burden of traditional cardiovascular risk factors than those with obstructive CAD [5,6,7]. However, among the traditional cardiovascular risk factors, the presence of hypertension, diabetes mellitus, dyslipidemia and smoking was noted [3,8].

The prevalence of diabetes mellitus (DM) worldwide has doubled in the last 30 years, from 7% to 14% [9]. If we also consider patients with impaired fasting glycemia and those with impaired glucose tolerance, conditions that characterize the transition between healthy status and diabetes, we will realize that this disease has already taken on a pandemic-like character. Vascular complications are the major manifestations of DM and are responsible for most of the morbidity, hospitalizations and mortality rates that occur in these patients. Microvascular complications include diabetic retinopathy, neuropathy, nephropathy, and coronary microvascular dysfunction. Macrovascular complications include epicardial coronary, cerebrovascular, and peripheral artery disease.

Patients with DM face not only early formation and accelerated progression of atherosclerotic plaques but also an increased risk of plaque destabilization and major adverse cardiovascular events (MACEs) [10]. In light of this reality, anginal symptoms and acute coronary events in patients with diabetes have been linked to coronary atherosclerotic burden. However, angiographic evaluation of the coronary arteries has often shown non-obstructive lesions, highlighting a discrepancy between the clinical manifestations and/or laboratory test results and/or ECG changes on the one hand and the objective angiographic evaluation on the other hand, suggesting that other mechanisms are also involved. Thus, it has been hypothesized that epicardial coronary artery spasm and various microcirculatory abnormalities may be contributors to MACEs in patients with diabetes.

Our review aims to perform an in-depth analysis of the available literature to provide up-to-date and comprehensive knowledge on coronary artery spasm in diabetes patients. As far as we know, this is the first article to focus exclusively on characterizing the relationship between coronary spasm and diabetes mellitus. It synthesizes, structures, and integrates data from studies published over the past quarter of a century. The objective is to provide reliable knowledge that allows for the optimization of clinical practice. Our search was conducted in the Web of Science database. The keywords used were “coronary spasm” or “epicardial spasm” or “microvascular spasm” or “microvascular dysfunction” or “vasospastic angina” or “microvascular angina” and “acetylcholine” or “ergonovine”. Only articles presenting results of original studies were retrieved. These were manually reviewed to identify whether they presented data on diabetes patients. The resulting articles were studied in extenso, and comparative data between patients with and without diabetes were extracted in tabular form. In order for our analysis to be robust and anchored in modern practice, we restricted the search to articles published in English since 2000.

## 2. Principles of Coronary Circulation Function Testing

The coronary vasculature has two major components. Macrocirculation is represented by the epicardial coronary arteries, which are large-caliber conductance vessels. Microcirculation includes medium and small-caliber vessels. Pre-arterioles and arterioles are resistance vessels and are responsible for the dynamic regulation and distribution of coronary blood flow, accounting for 80% of coronary flow resistance [11]. Capillaries are exchange vessels and contribute to another 15% of the coronary flow resistance. The remaining resistance is mainly located in post-capillary venules and small veins.

Impairment of coronary macro- and/or microcirculation has consequences on myocardial perfusion and can lead to myocardial ischemia during activities—from high-demand events to personal care gestures—or at rest. Blood flow limitation in the epicardial coronary arteries can be the consequence of atherosclerosis (fixed obstruction) or epicardial vasospasm (dynamic obstruction). Other causes, such as muscular bridging or spontaneous dissection, are rarer etiologies [1]. Blood flow limitation in the microcirculation has a broader range of contributing mechanisms, grouped under the umbrella of coronary microvascular disease [12]. Structural and functional abnormalities of the vascular wall and extravascular compression are the main substrates of coronary microvascular disease. In real-life conditions, multiple mechanisms act simultaneously and have a synergistic effect.

To confirm the diagnosis of coronary microvascular dysfunction, evaluation of coronary circulation function along with the exclusion of obstructive lesions of the epicardial coronary arteries are necessary. Among non-invasive tests, such as PET/CT or PET + CT coronary angiography, only hybrid techniques that combine epicardial coronary artery imaging with coronary microcirculation function testing in a single test have this capability. Current guidelines highlight the possibility of performing invasive coronary circulation function testing in a single procedure with angiography, which is an investigation with much wider availability [2]. By following this algorithm, a complete assessment of the coronary circulation can be performed. Anatomic and hemodynamic data will be obtained, and vasomotor function will be tested.

Functional abnormalities present as impaired vasodilation and/or increased vasoconstriction. Invasive coronary circulation function testing includes assessment of endothelium-dependent vasodilation (endothelial function), endothelium-independent vasodilation (vasodilatory capacity of the coronary circulation), and vasospasm provocation testing [13]. A vasoreactivity protocol based on intracoronary administration of acetylcholine (Ach) and adenosine has been proposed [2] to enable the complete assessment of coronary function and to differentiate between macro- and microcirculatory impairments, and between endothelium-dependent and endothelium-independent mechanisms, respectively.

Administration of ACh begins with a 2 μg dose and continues with 20 μg, 100 μg, and 200 μg doses. Low doses (2 μg to 20 μg) of ACh allow for the assessment of endothelial function. At the endothelial level, ACh triggers the release of relaxing factors such as nitric oxide (NO)—a molecule with a strong vasodilating effect. At the level of vascular smooth muscle cells (VSMCs), ACh has a vasoconstrictor effect [13]. In the healthy endothelium, the vasodilatory effect dominates, resulting in coronary vasodilation. Thus, upon ACh administration, epicardial vasodilation and increased blood flow in coronary microcirculation are expected. If endothelial dysfunction is present, the vasodilatory capacity is impaired and mild vasoconstriction occurs, with a reduction in epicardial coronary artery diameter of less than 90%. At the microcirculation level, an abnormal flow response is identified, namely the increase in coronary blood flow is less than 50% [5].

High doses (100 μg to 200 μg) are used to induce coronary spasm. Coronary spasm is defined as intense, reversible vasoconstriction of the coronary arteries that results in total or subtotal occlusion of the vessels, leading to impaired myocardial perfusion [14]. The resulting myocardial ischemia may be symptomatic—manifested as stable angina, acute coronary syndromes (ACSs), life-threatening ventricular arrhythmias or sudden cardiac death—or asymptomatic (silent ischemia), reflected by transient ST-segment elevation or depression on resting ECG. Research advances have confirmed that spasm can occur in both normal and atherosclerotic vessels and can affect the epicardial coronary arteries and the coronary microcirculation, either individually or together [15]. Thus, an ACh-induced epicardial spasm test can be positive for focal spasm, defined as transient, total, or subtotal focal occlusion (>90% stenosis) of a coronary artery associated with both symptoms and signs of myocardial ischemia (anginal pain and ischemic ECG changes) or for diffuse spasm, defined as 90% diffuse vasoconstriction induced in ≥2 contiguous segments of a coronary artery associated with both symptoms and signs of myocardial ischemia (anginal pain and ischemic ECG changes) [16]. Microvascular spasm is presumed when anginal pain and ischemic ECG changes occur with ACh administration in the absence of visible severe epicardial spasm.

At the end of the ACh-induced spasm test, 200 μg of intracoronary nitroglycerine is routinely administered to revert any remaining coronary spasm [5]. Then, vasodilator function testing is performed using 200 μg of adenosine [2]. The aim is to assess the endothelium-independent reserve vasodilator capacity of the coronary circulation. An abnormal response is characterized by low coronary flow reserve and high microcirculation resistance.

Another compound widely used in clinical practice to induce coronary spasm is ergonovine (ER). The Coronary Vasomotion Disorders International Study Group (COVADIS) statement emphasizes that intracoronary ACh is usually used, but intracoronary or intravenous ER is a valid alternative [17].

## 3. The Common Ground of Diabetes Mellitus and Coronary Artery Spasm

**Figure 1:**
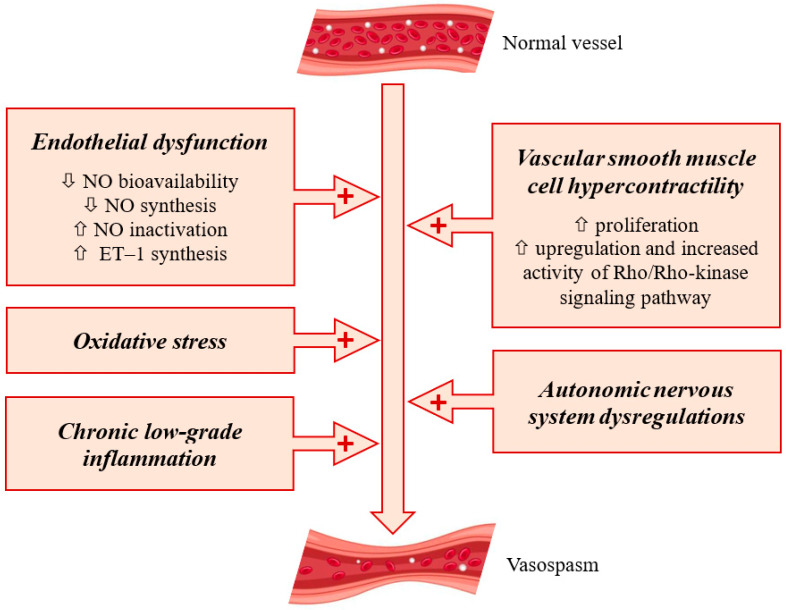
Major determinants of coronary spasm. The arrows show that endothelial dysfunction, vascular smooth muscle cell hypercontractility, oxidative stress, autonomic nervous system dysregulations, and chronic low-grade inflammation contribute to the occurrence of coronary spasm.

The substrate of coronary artery spasm is complex and still incompletely elucidated despite major advances in the invasive assessment of vascular reactivity. Endothelial dysfunction, oxidative stress, VSMC hypercontractility, autonomic nervous system dysregulations, and chronic low-grade inflammation are situated at the crossroads between DM and coronary artery spasm (Figure 1).

### 3.1. Endothelial Dysfunction

Endothelium mediates both vasodilation and vasoconstriction. The substances with vasodilatory effect produced by the endothelium are NO, which is the crucial vasodilator, prostaglandins, such as prostaglandin E1 and prostacyclin, and endothelium-derived hyperpolarizing factor (EDHF) [18]. The main vasoconstrictors produced by the endothelium are endothelin-1 (ET-1) and angiotensin II (Ang II). Endothelial dysfunction is present in patients with DM and is characterized by an imbalance between vasodilators and vasoconstrictors, which favors vasoconstriction, mainly determined by decreased NO synthesis, NO inactivation, and increased ET-1 generation [19,20].

The relationship between reduced NO bioavailability and coronary spasm has long been proven. Administration of L-arginine, a precursor of NO, has the ability to attenuate ACh-induced coronary vasospasm [21,22]. Experimental and clinical studies have shed light on ET-1’s contribution to coronary spasm [23,24,25,26]. Patients with ACh-induced coronary spasm had higher ET-1 levels than non-spasm patients both at baseline and during a provocation test [26]. Furthermore, vasoconstriction in response to ET-1 was greater both in patients with ACh-induced epicardial spasm and in those with microvascular dysfunction, compared with patients with a negative provocation test [25].

Genetic evidence, such as polymorphisms of the *eNOS* and *ET-1* genes, also supports the association between endothelial disfunction and coronary spasm [27]. A Japanese study focusing specifically on the relationship between *eNOS* gene polymorphism and coronary spasm identified a significant association between spasm and three point mutations in the 5′-flanking region of the *eNOS* gene, namely a T-to-C mutation at nucleotide position −786, an A-to-G mutation at nucleotide position −922, and a T-to-A mutation at nucleotide position −1468 [28]. The study also showed that the mutant allele of the *eNOS* gene was a better predictor of spasm than traditional cardiovascular risk factors. Given that NO regulates the production of ET-1, patients with a mutated *eNOS* gene have not only reduced NO production but also increased ET-1 production, both of which contribute to the onset of spasm. A study evaluating the *ET-1* gene polymorphism in relation to coronary spasm in a Western European population found that the rs9349379-G allele is associated with higher ET-1 levels and more than doubles the risk of coronary microvascular dysfunction [29].

### 3.2. Oxidative Stress

Oxidative stress is an imbalance between the production of reactive oxygen species (ROS) and antioxidant defense, ending in cellular damage and dysfunction [30]. In patients with DM, the production of ROS is markedly increased due to persistent hyperglycemia. Several mechanisms are involved, namely glucose auto-oxidation, activation of the polyol pathway, production of advanced glycation end-products (AGEs), and mitochondrial dysfunction [31]. Excessive ROS production may contribute to vasospasm through several mechanisms, such as NO degradation, leading to a decrease in its bioavailability, reduced prostacyclin production, increased ET-1 production, uncontrolled proliferation of VSMCs and fibroblasts, increased inflammatory responses, and promotion of atherosclerosis [20,30,32].

Oxidized LDL is an established marker of ROS activity and has been related to coronary spasm in several studies. Patients with vasospastic angina had higher plasma levels of oxidized LDL than controls [33]. In addition, increased levels of oxidized LDL were an independent and powerful determinant of vasomotor dysfunction in patients with chest pain or myocardial ischemia on ECG and normal coronary arteries on angiography. In these patients, oxidized LDL levels correlated negatively with epicardial and resistant coronary vasodilator responses to bradykinin [34]. Furthermore, malondialdehyde-modified LDL, a form of oxidized LDL, is also associated with vasospastic angina [35].

### 3.3. Vascular Smooth Muscle Cell Hypercontractility

Hyperglycemia, directly or through AGEs—glycated and oxidized proteins and lipids—interferes with the function of several ion channels and metabolic pathways, leading to abnormal regulation of basal myogenic tone and VSMC hyperreactivity [36,37]. Among VSMC ion channels, there is increased L-type Ca^2+^ channel activity and significantly amplified Ca^2+^ influx, which leads to increased cytosolic calcium levels, which promote VSMC contraction. K^+^ channels are major regulators of VSMC membrane potential and intracellular Ca^2+^ concentration. Chronic hyperglycemia reduces K^+^ channel activity, favoring vasoconstriction [37]. Furthermore, AGEs activate protein kinase C (PKC) and Rho/Rho-kinase signaling pathways, which leads to the inhibition of myosin light chain (MLC) phosphatase and calcium sensitization [36]. It should be noted that MLC phosphorylation leads to contraction, while dephosphorylation by MLC phosphatase leads to relaxation. Thus, inhibition of MLC phosphatase function determines impaired relaxation. In addition, activation of PKC is associated with increased force and slowed relaxation of VSMC [38]. Diabetic VSMCs may be hyperresponsive to constricting signals, such as ET-1, angiotensin II, and may react more intensely and for a longer period of time. In addition, the number of vasoconstrictor receptors may be upregulated, worsening constriction.

There is increasing evidence supporting the essential role of upregulation and increased activity of Rho-kinase in the pathogenesis of coronary artery spasm [26]. In an animal model of coronary artery spasm, the upregulation of Rho-kinase was identified in spastic segments of coronary arteries. In both animal and human studies, it was demonstrated that Rho-kinase inhibitors dose-dependently attenuate or prevent coronary vasoconstriction [39,40]. Furthermore, Rho-kinase inhibitors ameliorated myocardial ischemia in patients with microvascular dysfunction [41] and in those with microvascular angina attributable to coronary microvascular spasm [42]. In addition, genetic testing has found that mutations in Rho-family GTPases, such as the Ala370Ser mutation in Rho GTPase-activating protein 9, can lead to coronary spasm [43]. Other mutations in genes involved in VSMC contractility, such as Rho-associated kinase 2 (ROCK2), variant aldehyde dehydrogenase 2 (ALDH2*2), and the East Asian-specific RNF213 variant, have also been associated with increased susceptibility to coronary spasm [44].

It has been hypothesized that coronary arteries with spontaneous or induced vasospasm exhibit increased basal vascular tone [45,46]. OCT studies provided confirmatory evidence by showing that abnormal medial contraction was present even when the artery was in an asymptomatic state [47]. A larger medial surface area and increased medial thickness at baseline characterized the vasospastic segments.

### 3.4. Autonomic Nervous System Dysregulations

Vessel walls are innervated by the sympathetic and parasympathetic nervous systems. Sympathetic nerves end at the level of the muscular layer, and their stimulation leads to vasoconstriction. Parasympathetic nerves are coupled with the muscarinic M3 receptors on the endothelium, leading to vasodilation by stimulating the NO pathway, and with muscarinic M3 and M2 receptors on the muscular layer, leading to vasoconstriction [48]. If the endothelium is healthy, the overall resulting effect is vasodilation, but if there is endothelial dysfunction, a paradoxical effect of vasoconstriction occurs.

Diabetic autonomic neuropathy is a common chronic complication of the disease and is the consequence of neuronal injury induced by the hyperglycemic state. The parasympathetic nervous system is affected first, resulting in unbalanced sympathetic activity. Thus, a relative increase in the sympathetic tone occurs [49].

The well-documented circadian variation in the occurrence of spasm (at rest, from midnight to early morning) and in the exercise capacity of patients with spasm (spasm induced by effort in the early morning, but not in the afternoon) suggests that autonomic nervous system contributes to coronary vasospasm [50]. An early study demonstrated that elevated vagal tone and increased sensitivity to adrenergic stimulation may trigger coronary spasm [51]. A recent study evaluated the relationship between heart rate recovery (HRR) after exercise, a marker of autonomic nervous system dysfunction, and the occurrence of spasm. In the first minute after exercise, the heart rate usually drops 18 beats/min or more. A value ≤ 12 beats/min suggests slow heart rate recovery after exercise and is an index of decreased parasympathetic activity. The study found that HRR was lower in patients with versus without spasm; HRR was significantly associated with the extent of coronary spasm, and a slow HRR was an independent predictor of spasm [52].

### 3.5. Inflammation

Chronic low-grade inflammation accompanies diabetes from the inception of metabolic imbalance to the development of complications [53]. Visceral white adipose tissue is the main source of inflammatory molecules, synthesizing and releasing a wide range of bioactive substances. Many adipokines, such as tumor necrosis factor alpha (TNF-α), and interleukins (IL), such as IL-1, IL-6, IL-10, are key players in inflammatory pathways [54]. One of the most important inflammatory pathways activated in diabetes is the transcription factor NF-kappaB. Macrophages and immune cells, namely B cells and T cells, can infiltrate adipose tissue and enhance the production of cytokines and chemokines, fueling local and systemic chronic low-grade inflammation.

There is a bidirectional relationship between inflammation and coronary artery spasm. On the one hand, inflammation can act as trigger for coronary spasm, and on the other hand, prolonged and repetitive coronary spasm can induce myocardial damage and more inflammation. Clinical studies identified increased plasma levels of several inflammatory markers in patients with coronary spasm. C-reactive protein (CRP), polymorphonuclear neutrophils and monocytes had higher levels in patients with spasm than in controls [55]. IL-6 and CRP were significantly higher in patients with vasospastic angina than in patients with stable CAD and normal controls, respectively [56]. High-sensitivity CRP (hs-CRP) had significantly higher levels in patients with spasm compared with those without spasm. In the same study, in multivariate analysis, hs-CRP was independently associated with coronary spasm, with an OR of 2.28 (*p* = 0.027) and with a cutoff of ≥2 mg/L [57]. CRP and sCD40L were correlated with the presence of both epicardial and microvascular spasm [58].

Some studies have delved deeper into the relationship between inflammation and coronary spasm by exploring local inflammation. Animal studies identified inflammatory cells in vasospastic segments [59]. Using advanced imaging techniques (CT coronary angiography and ECG-gated ^18^F-FDG PET/CT hybrid imaging), perivascular components were evaluated in relation to coronary spasm [60]. Inflammation in the adventitia and perivascular adipose tissue was found to be more prominent in patients with versus without spasm. Coronary perivascular ^18^F-fluorodeoxyglucose (^18^F-FDG) uptake and coronary perivascular adipose tissue volume were higher in patients with versus without spasm.

Endothelial dysfunction, oxidative stress, VSMC hypercontractility, autonomic nervous system dysregulations, and chronic low-grade inflammation are not independent pathways but key points in a complex network of metabolic processes. For instance, TNF-α and IL-1, markers of inflammation, upregulate the Rho/Rho-kinase signaling pathway, thereby influencing VSMC reactivity [32]. Inflammation suppresses endothelial function and NO activity, leading to endothelial dysfunction. Oxidized LDL activates innate and adaptive immunity, paving the pathway that links oxidative stress to inflammation [61].

## 4. Epicardial Spasm

Epicardial spasm was the most studied (Table 1 and Table 2). There is growing evidence that epicardial coronary spasm is commonly associated with atherosclerosis [62,63,64]. In a Korean study, 884 patients with angina and non-severe coronary artery stenosis underwent intracoronary ER testing [63]. Vasomotor responses were compared between two groups: patients with coronary stenosis of less than 30% (non-stenosis group) and stenosis between 30% and 69% (mild-to-moderate stenosis group). A third of patients (37.7%) had coronary artery spasm. The incidence of spasm was higher in the mild-to-moderate stenosis group (54.6%) compared to the non-stenosis group (34.2%, *p* < 0.001). As expected, patients in the mild-to-moderate stenosis group were older and had a significantly higher burden of traditional cardiovascular risk factors, including DM (*p* = 0.014), than those in the non-stenosis group. In a larger Korean study, over 6500 patients with angina and insignificant coronary artery stenosis underwent ACh testing [64]. Vasomotor responses were compared among three groups: patients with coronary stenosis of less than 30% (non-stenosis group), between 30% and 49% (mild stenosis group), and between 50% and 69% (moderate stenosis group). More than half of the patients (58.1%) had coronary spasm. The incidence of spasm was higher in the mild stenosis group (60.1%) and the moderate stenosis group (60.9%) compared to the non-stenosis group (55.3%, *p* < 0.001). Again, as expected, patients with mild and moderate stenosis were older and had a significantly higher burden of traditional cardiovascular risk factors, including DM, than those in the non-stenosis group. Considering that significant atherosclerosis multiplies the risk of spasm more than twice [63], patients with coronary atherosclerosis, such as those with diabetes, should be considered a vulnerable population. On the contrary, a Western European study showed that in patients with stable angina and non-obstructive coronary arteries, DM was not an independent predictor for epicardial or microvascular spasm [65].

A Japanese study showed that while epicardial stenosis ≥ 75% was an independent risk factor for a positive ACh-induced spasm test, DM was not (univariate analysis OR: 1.183; 95% CI: 0.924–1.515, *p* = 0.183) [79]. Significant data regarding coronary atherosclerotic burden, ACh-induced spasm and DM were provided by a large retrospective Japanese study [71]. DM correlated negatively with ACh-induced coronary spasm in patients with epicardial stenosis ≥ 75%. Simple logistic regression analysis showed an OR of 0.63 (95% CI: 0.40–0.99; *p* = 0.046). Multiple logistic regression analysis confirmed the lack of correlation, with an OR of 0.59 (95% CI: 0.35–0.99; *p* = 0.047) [71]. Furthermore, the site of spasm—at the site of organic stenosis versus at sites other than the site of organic stenosis—was not influenced by the presence of DM in patients with epicardial stenosis ≥75%. The authors of the study hypothesized that coronary spasm is more likely to occur in vessels with early-stage atherosclerosis than in vessels with advanced, heavily calcified plaques, as often found in long-term or uncontrolled DM.

In 2020, the most detailed analysis of the relationship between coronary spasm and atherosclerosis was published [67]. The overall assessment showed that atherosclerotic plaque frequency, diameter percent stenosis, and atherosclerotic burden were higher in patients with coronary spasm than in those without spasm, with the left anterior descending coronary artery (LAD) being the most affected. Patient-level analysis showed that the atherosclerosis of the spastic artery was always more important than that of the other arteries (higher plaque frequency, higher mean percent stenosis, and higher plaque burden). An interesting finding was that atherosclerotic plaque parameters were higher in patients with spasm, both in the spastic artery and arteries irrelevant to spasm, than in patients without spasm. It was hypothesized that the occurrence of vasospasm depends on the whole vascular atherogenic milieu, reflecting its systemic nature. This study also identified cut-off points of coronary stenosis as predictors of spasm. A LAD stenosis ≥ 35% and LCx stenosis ≥ 35% were independent risk factors for spasm. Furthermore, a composite parameter, LAD stenosis ≥ 35% or LCx ≥ 35% or RCA ≥ 40%, was also an independent risk factor for spasm. However, there was no difference in the prevalence of DM between patients with versus without ACh-induced spasm (*p* = 0.760).

A study on 75 patients with persistent angina in the absence of obstructive CAD provided highly refined and subtle information on the association between coronary spasm and atherosclerosis [80]. All patients underwent ACh testing to assess the occurrence of vasospasm. The Gensini score was used to quantify the extent and severity of CAD burden on angiography, allowing precise stratification of the degree of coronary artery stenosis. Optical coherence tomography (OCT) was used to assess atherosclerotic morphological characteristics. This study showed that, even in a population with a very low atherosclerotic burden, patients with spasm had more advanced atherosclerosis compared with patients without spasm (Gensini score 1 vs. Gensini score 0, *p* = 0.04) [80]. Patients with spasm had greater total plaque extension and more markers of plaque vulnerability than patients without spasm. Interestingly, none of these parameters differed between patients with epicardial and microvascular spasm, and there was no correlation between total plaque extension and the type of epicardial spasm—focal or diffuse. However, among markers of plaque vulnerability, the prevalence of intraplaque neovascularization differed significantly between patients with spasm (37%) compared with those without spasm (6%, *p* = 0.02), suggesting that the dysfunctional endothelium of these neoformation vessels, with an impaired NO pathway, predisposes to the occurrence of spasm [80]. Impaired microvascular dilation was identified in more than one-third of patients with coronary spasm (39%), emphasizing that vascular dysfunction is the result of the cumulative action of several mechanisms. Due to the low prevalence of DM in the enrolled population (8%), the study conclusion could not be further refined for this subgroup.

Significant information was also provided by a small study that simultaneously assessed coronary artery function—by ACh testing—and morphological changes in the vascular wall attributable to atherosclerosis—by intravascular ultrasound (IVUS) and near-infrared spectroscopy (NIRS)—in patients with chest pain and coronary stenosis <30% on angiography [85]. While no difference in atherosclerotic burden was evident between patients with and without epicardial coronary endothelial dysfunction on gray-scale IVUS imaging, NIRS clearly demonstrated the opposite. This discrepancy stems from the ability of NIRS to identify the lipid core of atherosclerotic plaques, which is a marker of active atherosclerosis. Thus, NIRS demonstrated the association between epicardial coronary endothelial dysfunction and an active atherosclerotic process, which is more significant than the association with atherosclerosis itself. It is well known that DM can change plaque morphology, transforming its phenotype into a vulnerable one (thinner fibrous cap, larger lipid-rich core, and increased macrophage accumulation) [86]. Thus, patients with diabetes, especially those with poor long-term glycemic control, are more prone to vulnerable atherosclerotic plaques than those without [87]. It can be hypothesized that in relation to spasm, the atheroma phenotype could be more important than the atheroma burden.

Coronary IVUS assessment combined with ACh testing showed that sections with normal and abnormal endothelial function coexist along the same coronary artery, with endothelial dysfunction taking on a segmental pattern [88]. Coronary segments with endothelial dysfunction—that responded to ACh by vasoconstriction—had more vulnerable atheromatous plaques, namely with necrotic core areas. Given that sites of active inflammation and oxidative stress reside in necrotic core areas, and that inflammation and oxidative stress favor atherosclerosis, this dysfunction loop self-perpetuates. Coronary segments showing macrophages and microchannels on OCT, an expression of inflammation and vasa vasorum proliferation found in early stages of coronary atherosclerosis, also had an abnormal response to ACh testing [89]. Vasa vasorum density negatively correlated with endothelium-dependent microvascular function assessed by ACh testing in patients with early coronary atherosclerosis [90]. In patients with INOCA, OCT demonstrated an increase in adventitial vasa vasorum regardless of the type of ACh-induced spasm [76]. Patients with diffuse spasm had a higher density of adventitial vasa vasorum compared with those with microvascular spasm. Patients with focal spasm had the worst atherosclerotic phenotype, with lipid-rich fibroatheroma and intraplaque neovessels. However, there was no difference in the prevalence of DM between subgroups [76].

Atherosclerosis and inflammation are tightly linked. Furthermore, coronary perivascular inflammation—including adventitia and perivascular adipose tissue—participates in the occurrence of spasm [60]. Although historically considered to have little contribution to normal vessel function, the adventitia is now recognized as an important structure in the etiology of spasm, as it mediates communication between vascular smooth muscle cells—whose hypercontraction is the organic substrate of spasm—and the adjacent perivascular adipose tissue. Dysfunctional perivascular fat, a common feature of diabetes patients, releases inflammatory mediators that promote angiogenesis and vasa vasorum proliferation [91]. The consequence is the amplification of atherosclerosis progression by increasing the trafficking of cells and inflammatory mediators from perivascular fat to the vascular wall and promoting intraplaque hemorrhage.

The bidirectional potentiation relationship between spasm and atherosclerosis is supported by much evidence. In short, repetitive mechanical insult through spasm can lead to plaque growth and destabilization, the latter possibly ending in ACS. Atherosclerotic plaque can be both a trigger and aggravating factor for vasospasm as it creates an environment characterized by endothelial dysfunction, low-grade inflammation, and VSMC hyperreactivity [67].

Identifying the coexistence of coronary spasm with atherosclerosis is particularly important for patient outcomes. The prevalence of plaque erosion and thrombosis, as well as thrombus size, were greater in segments with spasm than in segments without spasm [69]. Furthermore, a particular location of the thrombus was revealed by OCT, namely at the sites of spasm (77.4%). Of patients with vasospastic angina and non-obstructive coronary arteries, those presenting to the emergency department with ACS were younger and had a higher prevalence of DM compared with those presenting without ACS (*p* < 0.001) [92]. In the subgroup of patients with myocardial infarction, the prevalence of DM remained high (*p* < 0.001). Given that urgent presentation with confirmed myocardial ischemia reflects severe narrowing or occlusion of the coronary artery during vasospasm, it should be emphasized that diabetes patients with coronary spasm and non-obstructive coronary arteries are at increased risk of MACEs. In patients with more severe stenosis, namely epicardial stenosis ≥75%, spasm occurred more frequently at the site of organic stenosis compared with sites other than the site of organic stenosis, and spasm at site of organic stenosis was strongly associated with an increased risk of MACEs [71].

## 5. Microvascular Spasm

Microvascular spasm is increasingly recognized and studied as an important component of coronary microvascular dysfunction. The presence of microvascular spasm has clinical implications, as it is an independent predictor for recurrent angina, thereby having the potential to affect long-term quality of life [81]. Systemic microvascular abnormalities seem to be common in patients with coronary microvascular dysfunction and/or epicardial vasospasm [25]. They share a common substrate, namely endothelial dysfunction and enhanced vasoconstriction. Functional abnormalities in peripheral small arteries were found in patients with coronary microvascular dysfunction and/or epicardial vasospasm [25]. In addition, a trend toward an association of microvascular spasm with the occurrence of stroke was recently identified [81].

There is great heterogeneity between studies regarding the prevalence of microvascular spasm. The ethnicity of the enrolled population (Caucasian vs. Asian), the female/male ratio in the study group, and the protocol used to induce spasm (ACh testing method, testing the other coronary artery, using a guidewire) are the main contributors to the variability in the results. While several studies reported a dominance of epicardial spasm over microvascular spasm [75,76,77,93], others reported the opposite [25,65,83,94,95]. The ratio of epicardial spasm to microvascular spasm was <1 in studies conducted on Caucasian populations and >2 in studies conducted on Asian populations [75]. The median ratio of epicardial spasm to microvascular spasm in Western studies was 0.68 (interquartile range: 0.19–0.83), significantly lower compared with that in Japanese studies, at 5.22 (interquartile range: 2.17–8.23), *p* < 0.05 [75].

There is increasing evidence of the coexistence of epicardial and microvascular spasm. It was shown that increased coronary vasoconstrictive reactivity, reduced coronary vasodilatation, and increased coronary microvascular resistance frequently coexist in various combinations in patients with angina and non-obstructive CAD. Patients with ACh-induced epicardial spasm had a nearly threefold risk of having an increased microcirculatory resistance index (≥18.0) [77]. This study concluded that epicardial spasm was an independent predictor for a high microcirculatory resistance index. Furthermore, the coexistence of ACh-induced epicardial spasm with a high microcirculatory resistance index increased the risk of MACEs by six times compared with the absence of both. Two studies in which the incidence of microvascular spasm was higher than that of epicardial spasm reported that 7% [94] and 21% [95] of patients had spasm in both territories, respectively. A Japanese study in which the incidence of microvascular spasm was very low compared to that of epicardial spasm reported that only four patients had both epicardial and microvascular spasm [75]. A recent study evaluating patients with INOCA by OCT and ACh stimulation showed that microvascular spasm coexisted in 70% of diffuse spasms and 68% of focal spasms [76].

While some parameters, such as female sex, are constantly associated with microvascular spasm [75,78,84], there are few data available on DM. In a cohort of 847 Caucasians who underwent ACh testing for suspected myocardial ischemia with non-obstructive coronary arteries, microvascular spasm was identified in a large number of patients (205 patients, 24.2%) [84]. However, only 34 (17%) had DM. Although it is well known that DM is a condition that impairs microvascular function, DM has not been found to be a predictor of microvascular spasm. Similar results were provided by a larger European study. In patients with angina and non-obstructive CAD, DM was not associated with either epicardial spasm or microvascular dysfunction, neither in univariate nor multivariate analysis [83]. The results of the Asian studies reinforce previous findings. Microvascular spasm was identified in 29.5% of 4644 patients with typical rest chest pain and non-obstructive CAD, and only 12.6% had diabetes [78]. The prevalence of DM was similar between the epicardial spasm, microvascular spasm, and non-spasm groups. A Japanese study showed that the incidence of microvascular spasm in the LCA territory was statistically significantly higher than that in the RCA territory (30 patients vs. 14 patients, *p*  <  0.001) [75]. In addition, 10 patients (25%) had microvascular spasms only in RCAs. However, DM was not associated with the presence of spasm, nor with its location, microvascular or at the level of epicardial coronary arteries.

## 6. Acetylcholine vs. Ergonovine Testing

There is extensive clinical experience on both substances. However, given the complex substrate of coronary artery spasm in diabetes patients and the fact that one size may not fit all, the question arises of whether there should be a preference. Different mediators may have the potential to lead to different responses [73].

ACh is a major neurotransmitter of the parasympathetic nervous system. It has multiple sites of action and complex biological effects. At the coronary level, the effects result from the interaction of ACh with the M3 subtype of the muscarinic receptor, which is a G protein-coupled receptor [96]. The binding of ACh to the endothelial M3 receptor activates the Gq protein, which further activates endothelial phosphoinositol-specific phospholipase C, resulting in inositol triphosphate (IP3) and diacylglycerol production. IP3 triggers the release of intracellular calcium from internal stores. The increased calcium level activates endothelial nitric oxide synthase (eNOS), an enzyme that converts L-arginine to NO. NO diffuses into adjacent VSMCs and increases the activity of soluble guanylyl cyclase and the concentration of cyclic guanosine monophosphate, thereby promoting relaxation. While the effect is mediated by NO at the endothelial level, at the VSMC level, the effect is direct. The binding of ACh to the M3 receptor of VSMCs leads to an increase in intracellular calcium, which activates myosin light-chain kinase, leading to actin–myosin interaction and VSMC contraction. The outcome at the whole-vessel level depends on endothelial function [97]. A healthy endothelium is able to produce sufficient NO to override the direct vasoconstrictor effect of ACh on the VSMCs, resulting in vasodilation. When the endothelium is dysfunctional, NO release is impaired, so the vasoconstrictor effect of ACh on VSMC becomes dominant, paradoxically resulting in constriction instead of dilation.

Ergonovine is an agonist for serotonergic receptors (5-HT) and alpha-adrenergic receptors (α-AR). Mediation of serotonin-induced contraction in coronary arteries is primarily mediated by 5-HT1 receptors—particularly 5-HT1B and 5-HT1D subtypes—and secondarily by 5-HT2 receptors [98]. They are G-protein-coupled receptors. Activation of the 5-HT1 and 5-HT2 receptors triggers an intracellular signaling cascade, namely the phospholipase C-IP3 pathway, which leads to an increase in intracellular calcium levels. Calcium activates L-type voltage-gated calcium channels in the VSMCs, causing muscle contraction, which is reflected at the whole-vessel level by vasoconstriction. ARs play an important role in the regulation of coronary blood flow, with α-AR causing vasoconstriction and β-AR causing vasodilation [99]. α-AR are members of the G-protein-coupled receptors. Upon activation, they trigger the intracellular phospholipase C-IP3 pathway, leading to an increase in intracellular calcium levels, VSMC contraction, and vasoconstriction. Activation of α1-ARs elicits a response in epicardial coronary arteries and large arterioles, whereas activation of α2-ARs elicits a response in the coronary microcirculation.

In summary, ACh has dual opposing effects on the coronary artery. It causes endothelium-dependent vasodilation and endothelium-independent constriction of VSMC. ER causes endothelium-independent constriction of VSMCs. Some researchers hypothesized that VSMC hyperconstriction, mediated by Rho-kinase activation, has a major contribution to coronary spasm, challenging the widely accepted dominant role of endothelial dysfunction [100]. Thus, ACh-induced coronary spasm is the combined effect of endothelial dysfunction and VSMC hyperconstriction. ER activates 5-HT receptors; thereby, the ER-induced coronary vasoconstricting response mainly represents endothelium-independent VSMC hyperconstriction. However, it is conceivable that the effect of ER could be enhanced by a dysfunctional endothelium [67].

This data can explain some clinical observations.

Several studies compared the coronary artery response to ACh and ER [68,73,101]. One study reported that the frequency of spasms induced by ACh was not different from that induced by ER, neither in the overall analysis nor in the subgroup analysis according to the following variables: angina at rest, angina on exertion, angina at rest or on exertion, myocardial infarction, after percutaneous coronary intervention, atypical chest pain, valvular heart disease, dilated cardiomyopathy, and hypertrophic cardiomyopathy, respectively. Therefore, it was hypothesized that from the perspective of clinical background, any agent that induces spasms can be used [73]. Other studies reported low concordance of spasm sites and spasm configurations in the same coronary artery between ACh and ER [68,101]. Spasm non-concordance was higher in the RCA territory than in the LAD and LCx territories, respectively [101]. In addition, when ACh and ER tests were performed sequentially during the same angiography, more patients were positive for spasm [68,102]. One in ten patients who were negative for spasm on ACh testing became positive on ER testing. New positive spasms were identified in both the RCA territory (10.3%) and the LCA territory (7.4%) [68]. The new hypothesis was that the coronary artery tree might have some dominance of each receptor on both coronary arteries and that the supplementary use of ACh and ER might provide additional diagnostic benefits and should be used whenever possible [66,68]. It is recommended that sequential tests begin with ACh [68].

Another important aspect is the type of spasm they produce. ACh provokes spasms more diffusely and more distally than ER [68,73,101]. ER provokes spasms more focally and more proximally than ACh [68,73,101]. Furthermore, ER results in a significantly higher incidence of total occlusive spasm compared with ACh [72]. It was hypothesized that ER may cause higher coronary vasomotility than ACh [101].

Patients with diabetes face early and rapidly progressive atherosclerosis [10], and the atherosclerotic burden may influence the vascular response to the administration of the spasm inducer. On the one hand, more spasms in patients without fixed stenosis (*p* < 0.01) and more multiple spasms (*p* < 0.01) were observed with ACh than with ER [73]. This data is consistent with the results of another study that demonstrated that, by using the ACh provocation test, endothelial dysfunction could be identified at a stage when atherosclerotic lesions were not detectable by any imaging technique [103]. On the other hand, ER induced more spasms in patients with ≥75% lesions than in those with <75% lesions (*p* < 0.01) [73]. Recently published data from a multicentre study using ER as a spasm-inducing agent indicated a cutoff value of 35–40% of coronary stenoses, above which there was a strong correlation between atherosclerotic burden and spasm [67]. Thus, it can be hypothesized that the ACh provocation test might be more valuable in diabetes patients with angiographically normal coronary arteries or early-stage atherosclerosis (considering the ability of ACh to unmask endothelial dysfunction), whereas the ER provocation test might be more valuable in those with advanced atherosclerosis. Indeed, early research has shown that coronary arteries are more sensitive to serotonin at the level of organic stenoses [104]. Interesting results are provided by a comparative study between ACh and ER that included traditional cardiovascular risk factors in the analysis [73]. While smoking and dyslipidemia were statistically significantly associated with coronary spasm (*p* < 0.01) regardless of the spasm-inducing agent used, DM was associated with coronary spasm only when ER was used (*p* < 0.05).

ACh has a short half-life of only a few seconds. ER has a long half-life and, consequently, a longer duration of efficacy than ACh. As a consequence, ER-induced coronary spasm is less likely to resolve spontaneously than ACh-induced spasm [101]. The necessity for intracoronary administration of nitroglycerine to relieve coronary spasms before performing another coronary artery test was higher with ER than with ACh (*p* < 0.01) [73]. Furthermore, there have occasionally been situations in which ER-induced coronary spasm was refractory to intracoronary vasodilators [101].

Intracoronary administration of ACh has a high sensitivity (90%) and specificity (99%) for the diagnosis of vasospastic angina [105]. ER has a higher sensitivity but a lower specificity than ACh [62]. Furthermore, women are more sensitive to ACh than men and achieve a positive test at lower doses of ACh [83]. This finding was valid across the entire ACh dose range between 20 and 200 μg.

Both substances have an acceptable level of safety for clinical use [68,73,106]. Still, spasm provocation tests are not without risks. Therefore, intracoronary administration of ACh or ER should be performed prudently. Arrhythmic complications are the most common and occur in up to 6.8% of patients tested [66,72]. Almost half of them are major ventricular events, namely ventricular tachycardia (VT) and ventricular fibrillation (VF). Of note, in patients with vasospastic angina, the presence of DM was not correlated with either the occurrence of ST-segment changes during the test or the occurrence of provocation-related malignant ventricular arrhythmias [72]. A significantly higher prevalence of arrhythmic complications occurred when using ACh (9.3%) compared to ER (3.2%) (*p* < 0.001). The difference came from ventricular arrhythmias—VT/VF (ACh 4.9% vs. ER 0.8%, *p* < 0.001) and bradycardia/sinus pause (ACh 3.6% vs. ER 0.4%, *p* < 0.001) [72]. Wide QT dispersion was associated with increased risk of ventricular arrhythmias during the provocation test in patients with vasospastic angina [107]. ACh could affect QT dispersion because it has non-uniform effects on ventricular endocardial and epicardial action potential. This aspect might be significant in patients with DM complicated by neuropathy because increased QT dispersion is associated with diabetic cardiac autonomic neuropathy [108]. Therefore, ECG evaluation aiming to identify repolarization heterogeneity should be performed in all diabetes patients before the spasm test. As a wide QT dispersion of >120 ms increases the risk of VT, the medical team should ensure that the potassium level is in the upper normal range [109] and that appropriate medications and a defibrillator are available in the catheterization laboratory for emergency treatment. Given that the sinus and atrioventricular nodes are perfused by the RCA, high-dose ACh administered into the RCA can cause transient but severe brady-arrhythmia, frequently associated with a significant drop in blood pressure [110]. In patients successively tested with ACh and ER, arrhythmias were still the most common complication. One study reported that paroxysmal atrial fibrillation was recorded in 22.16% of the patients tested, while ventricular arrhythmias occurred in 0.95% of the patients tested [68].

## 7. Lessons Learned and Perspectives

DM and coronary artery spasm share several underlying pathophysiological mechanisms, such as endothelial dysfunction, oxidative stress, vascular smooth muscle cell hypercontractility, autonomic nervous system dysregulation, and chronic low-grade inflammation. Although there is strong plausibility for an association of spasm with DM, studies have failed to demonstrate that DM is an independent predictor of pharmacologically induced spasm. DM has not been consistently associated with either epicardial or microvascular spasm across studies.

Studies that evaluated epicardial spasm exclusively (Table 1) were conducted only in Asian patients (Japan, Korea, and Taiwan). Although all enrolled patients were symptomatic, there was a wide range of clinical presentations, from typical or atypical angina-like chest pain to successfully resuscitated out-of-hospital cardiac arrest. Some patients had high clinical suspicion of coronary spasm. Patients had non-obstructive CAD, except for one study in which spasm testing was also performed in patients with ≥75% organic stenosis on angiography [71].

The agent used for the spasm provocation test was either ACh or ER, except for two studies that used a sequential protocol, namely ACh followed by ER [68,72]. Despite this heterogeneity, individual study results pointed in the same direction, namely that DM is not an independent risk factor for spasm. Only one study showed the opposite results, namely more spasm in patients with diabetes than in those without, but only in patients tested with ER [73]. Furthermore, DM was not correlated with either the type of spasm—focal or diffuse—or the location of the spasm.

Studies that assessed both macrovascular and microvascular function in patients with diabetes were conducted in both Caucasian and Asian populations (Table 2). Patients had symptoms and/or signs of myocardial ischemia and non-obstructive CAD. The agent of choice for the spasm provocation test was ACh in all studies, except one that used ACh or ER [82]. No studies performed sequential testing. It is noteworthy that these studies were more uniform in terms of both inclusion criteria and the agent used for the spasm test. Again, the individual study results pointed in the same direction, namely that DM is not an independent risk factor for spasm, either macrovascular or microvascular.

It should be emphasized that endothelial dysfunction, oxidative stress, and chronic low-grade inflammation contribute to both coronary spasm and atherosclerosis. Patients with diabetes develop atherosclerosis earlier in life and experience more accelerated plaque progression and a vulnerable plaque phenotype [10,80,86,87,88]. There is evidence that when DM, atherosclerosis, and coronary spasm coexist, the risk of MACEs increases [69,71,92]. Therefore, it can be hypothesized that DM does not directly increase the prevalence of inducible spasm but rather acts as a modifier of the vascular substrate, thereby amplifying the clinical consequences of spasm when it occurs.

Angina with non-obstructive coronary arteries should not be viewed as a benign phenotype of ischemic heart disease. There is a well-documented association with decreased quality of life, frequent hospitalizations, repeated angiographies, and MACEs [111]. In patients with epicardial spasm, the Japanese Coronary Spasm Association (JCSA) score can be used for risk stratification. Scores ≥6 correlate strongly with a higher incidence of MACEs [112]. In patients with microvascular spasm/dysfunction, MACE prediction is based on functional assessment parameters. The most predictive are myocardial perfusion reserve index, index of microvascular resistance, coronary flow reserve, and coronary angiography-derived index of microvascular resistance. It was recently shown that microvascular disfunction strongly and independently predicts long-term adverse cardiovascular outcomes in patients with type 2 DM and non-obstructive CAD [113].

All this knowledge could have the power to change the way we approach the diagnosis and treatment of coronary artery spasm in diabetes patients. Given that the cluster of DM, atherosclerosis, and spasm increases the risk of MACEs, there should be no reluctance to perform spasm testing in diabetes patients with signs and/or symptoms of ischemia and non-obstructive CAD at the time of their first elective diagnostic coronary angiography. It should always be remembered that coronary artery spasm may be present regardless of atypical or silent symptoms [114].

Identifying coronary vasospasm will trigger treatment optimization [44,115]. We emphasize that most of the available evidence refers to epicardial spasm. Few drugs have been studied in relation to microvascular spasm and even fewer have shown benefits. Calcium channel blockers (CCBs) are the first-line therapy in patients with spasm. They reduce vascular tone and relieve spasm in both macro- and microcirculation [116]. If monotherapy fails to control symptoms, long-acting nitrates can be added to CCBs as second-line therapy. Short-acting nitrates are used only for angina attacks. Although nitrates are very effective in relieving spasm in the epicardial coronary arteries, they have virtually no effect on the microvasculature [117]. Nitrates are redundant in patients with microvascular spasm and should not be recommended. Beta-blockers should be avoided in patients with epicardial spasm. However, third-generation beta-blockers, such as nebivolol and carvedilol, improved symptoms in patients with microvascular angina [116]. Nicorandil is a vasodilator agent with effect in both the macro- and microvascular territories. However, due to the lack of prognostic benefits, nicorandil is currently indicated only in patients with refractory angina [111]. Fasudil prevented myocardial ischemia in patients with coronary macro- and microvascular spasm [40,42], but its use is not feasible due to limited availability—it is only available in injectable form and is not approved outside Japan. Cilostazol, a selective phosphodiesterase 3 inhibitor with vasodilator, antiplatelet, and anti-inflammatory effects, induced favorable effects on epicardial spasm and coronary microvascular function [118,119]. Statins represent an important therapeutic resource due to their pleiotropic effects. Beyond their LDL cholesterol-lowering effect, they have anti-inflammatory and antioxidant properties, reduce endothelial dysfunction, and prevent VSMC Rho-kinase activation [96]. Although statins are not currently standard therapy for vasospastic angina, their administration in diabetes patients as part of the treatment of CAD may provide additional beneficial effects on vasomotion. Fluvastatin given over CCBs reduced the rate of ACh-induced spasm compared with CCBs alone [120]. Coronary revascularization could be the last resort for patients with refractory spasm despite appropriate medical therapy. PCI might be indicated in patients with spasm at the site of the unstable focal atherosclerotic plaque [121,122].

In an era where the transition from personalized medicine to precision medicine is underway, interest in the genetic substrate of diseases is growing, as it will allow further optimization of treatment. A ROCK inhibitor—fasudil—could be a future treatment for patients with an alteration in the *ROCK2* gene. Administration of l-arginine or nitrate would be prioritized in patients with *eNOS* gene mutations [123]. Mutations in both the *eNOS* [28] and *ET-1* [29] genes are associated with increased circulating levels of ET-1, making the ET-1 receptor a therapeutic target of interest. To date, studies have not met expectations. While the ET-1 receptor antagonist atrasentan improved coronary microvascular endothelial function [24], macitentan did not reduce anginal burden in patients with epicardial or microvascular spasm [124]. A case report has highlighted the successful use of bosentan in a woman with refractory vasospastic angina [125]. Great hopes are now being placed in bosentan [126]. Identification of mutations in the *aldehyde dehydrogenase 2* gene that underlie the mechanism by which alcohol can trigger vasospasm may allow recommendations for lifestyle changes [127].

Our review has several limitations that stem from the wide heterogeneity of the studies analyzed. First, the enrolled patients reflect two distinct populations, Asians and Caucasians, which are historically known to differ in the prevalence and characteristics of coronary spasm. The higher frequency of spasm in Asians has been attributed both to higher smoking rates and to genetic characteristics. Specific polymorphisms in the *aldehyde dehydrogenase 2* and *ROCK2* genes have been associated with vasospasm only in Asians, not Caucasians [128,129]. However, emerging data from contemporary studies suggest that Asians and Caucasians may be comparable in terms of spasm types and frequency of multivascular spasm, opening the pathway for further research [130]. Second, great heterogeneity was identified in the criteria used to define spasm and in the laboratory protocols—the agent used to induce spasm, the protocol for administering the agent, and the technique of coronary instrumentation. All of these may influence the results and hinder their general comparison and interpretation. Studies that follow standardized protocols are certainly needed. Third, data collection on coronary artery spasm in patients with diabetes has been difficult. Studies that included small numbers of patients had small numbers of patients with diabetes, and statistical analysis was not possible. DM was always reported as a binary parameter. Therefore, details such as duration of DM, glycemic control (HbA1c), and microvascular complications are missing. Recent evidence confirms that longer duration of DM increases the risk of MACEs in patients with non-obstructive CAD [131]. Similarly, uncontrolled DM increases the risk of MACEs by 28% [132]. Therefore, studies that assess more diabetes-specific parameters are needed so that the characteristics of spasm can be correlated with the type, duration, control, complications and treatment of DM.

## 8. Key Points for Clinical Practice

1. Diabetes mellitus provides a substrate compatible with the occurrence of both epicardial and microvascular spasm.
2. Coronary atherosclerosis develops early in patients with diabetes, and in conjunction with the fact that atherosclerotic plaques occupying approximately 1/3 of the lumen could be predictive of spasm, there should be no reluctance to test for spasm in these patients.
3. The association between microvascular disease and epicardial spasm severely increases the rate of MACEs. Even though diabetes has not been shown to be an independent risk factor for coronary spasm, the association of the two entities must be considered highly dangerous. As the macrovascular and microvascular circulation can frequently be involved concurrently, rapid and accurate diagnosis and appropriate treatment are mandatory to improve patient outcomes.
4. Supplementary use of ACh and ER might provide additional diagnostic benefits in selected cases.

## Figures and Tables

**Table 1:** Original studies providing data exclusively on epicardial coronary artery spasm in diabetes patients.

Author, Year, Country	No, Mean Age ± SD, M (%) of Patients Undergoing a Spasm Test	Characteristics of the Population (Focus on the Coronary Tree)	Agent Used to Induce Spasm	Main Results on the Association Between Coronary Spasm and DM
Sueda et al. 2022 Japan [66]	680 64 ± 11 y 464 (68%)	Angina-like chest pain and non-obstructive CAD; coronary stenosis < 50% on angiography.	ACh	DM in patients with vs. without spasm 55 (18%) vs. 79 (21%) (*p* = 0.2386); no differences in the type of spasm among patients with DM; diffuse 29 (19%) vs. focal 14 (16%) vs. combined 12 (16%).
Jo et al. 2020 Korea [67]	2703 55.1 ± 11.3 y (spasm positive) 54.7 ± 13.0 y (spasm negative) 1477 (54.6%)	Suspected variant angina.	ER	DM in patients with vs. without spasm 175 (9.5%) vs. 86 (9.9%); (*p* = 0.760).
Sueda et al. 2019 Japan [68]	528 64.5 ± 11.4 y 333 (63.1%)	Spasm provocation tests whenever possible; no significant left main stenosis (≤50% narrowing); no triple-vessel disease; No two-vessel disease with total occlusion.	ACh followed by ER	DM did not correlate with the stress test results; RCA: ACh+ (16; 19.3%) vs. ER+ (6; 17.1%) vs. ACh+ and ER+ (7; 16.3%) vs. ACh+ or ER+ (29; 18.0%) vs. ACh− or ER− (56; 17.8%) LCA: ACh+ (13; 13.8%) vs. ER+ (9; 32.1%) vs. ACh+ and ER+ (7; 14.0%) vs. ACh+ or ER+ (29; 16.9%) vs. ACh− or ER− (75; 21.6%).
Shin et al. 2017 Korea [69]	132 55.0 ± 10.0 y 96 (72.7%)	Suspected vasospastic angina; coronary stenosis < 50% on angiography.	ER	DM in patients with vs. without spasm 7 (7.5%) vs. 8 (20.5%); (*p* = 0.060).
Komatsu et al. 2016 Japan [70]	47 43.3 ± 13.9 y 44 (94%)	Survivors of out-of-hospital cardiac arrest, fully recovered; no organic heart disease; no significant coronary stenosis on angiography (<75% narrowing); suspected CAS or VA.	ACh	DM was the only clinical characteristic with statistically significant difference between the studied groups (spasm only, VA only, both positive, both negative, *p* = 0.046); no patient with spasm had DM.
Ishii et al. 2015 Japan [71]	1760 63.0 ± 11.0 y 903 (51.3%)	Typical or atypical angina-like chest pain.	ACh	DM in patients with organic stenosis ≥ 75% on angiography, with vs. without spasm 73 (31.3%) vs. 52 (41.9%) (*p* = 0.045); DM in patients without organic stenosis on angiography, with vs. without spasm 106 (16.7%) vs. 116 (15.3%) (*p* = 0.466); DM in patients with spasm at site of organic stenosis vs. with spasm at sites other than site of organic stenosis 61 (31.8%) vs. 12 (29.3%) (*p* = 0.754).
Takagi et al. 2013 Japan [72]	1244 66 y ^a^ 938 (75.4%)	Vasospastic angina.	ACh or ER or both	DM did not influence the occurrence of ST-segment change during test (without vs. with): 154 (17%) vs. 49 (20%) (*p* = 0.37); DM did not influence the occurrence of provocation-related VT/VF (with vs. without): 9 (23%) vs. 203 (17%) (*p* = 0.35).
Sueda et al. 2004 Japan [73]	1508 873—Ach 63.7 ± 8.8 y 600 (68.7%) 635—ER 64.5 ± 10.1 y 399 (62.8%)	First diagnostic coronary angiography; no significant left main stenosis (≤50% narrowing); no triple-vessel disease; no two-vessel disease with total occlusion.	ACh or ER	DM in patients with vs. without spasm Ach: 48 (15.3%) vs. 88 (15.7%) ^b^ ER: 40 (21.2%) vs. 64 (14.3%) (*p* < 0.05).
Sueda et al. 2003 Japan [74]	42 62 ± 10 y 38 (88%)	Vasospastic angina; no significant left main stenosis (≤50% narrowing); no triple-vessel disease; no two-vessel disease with total occlusion; no fixed stenosis > 25% after nitrate administration.	ACh	Prevalence of DM Focal (2) vs. diffuse (1) vs. no spasm (1); Proximal (1) vs. mid (2) vs. distal spasm (0).
Wang et al. 2002 Taiwan [62]	93 57 ± 14 y 55 (59%)	Suspected acute coronary syndromes; insignificant coronary stenosis on angiography (<50% narrowing).	ER	DM in patients with vs. without spasm 11 (28%) vs. 13 (23%) ^b^

ACh = acetylcholine; ACh+ = positive ACh test; ACh− = negative ACh test; CAD = coronary artery disease; CAS = coronary artery spasm; DM = diabetes mellitus; ER = ergonovine; ER+ = positive ER test; ER− = negative ER test; LCA = left coronary artery; RCA = right coronary artery; VA = vasospastic angina; VF = ventricular fibrillation; VT = ventricular tachycardia; ^a^ = data are presented as median; ^b^ = without statistical significance.

**Table 2:** Original studies providing data on both macrovascular and microvascular function in diabetes patients.

Author, Year, Country	No, Mean Age ± SD, M (%) of Patients Undergoing a Spasm Test	Characteristics of the Population (Focus on the Coronary Tree)	Agent Used to Induce Spasm	Type of Spasm Studied	Main Results on the Association Between Coronary Spasm and DM
Asian studies					
Sueda et al. 2022 Japan [75]	746 64 ± 11 y 492 (66%)	First diagnostic angiography for suspected myocardial ischemia without AMI; non-obstructive coronary arteries (coronary stenosis < 50% on angiography).	ACh	E Mv	DM in patients with vs. without spasm 70 (19%) vs. 79 (21%) (*p* = 0.4978); DM in patients with E spasm vs. E+Mv spasm vs. Mv spasm vs. ACh test unclassified vs. no spasm 60 (18%) vs. 1 (25%) vs. 9 (25%) vs. 42 (22%) vs. 37 (20%) ^a^.
Nishimiya et al. 2021 Japan [76]	329 NR 176 (53.5%)	INOCA (luminal narrowing < 50% on coronary angiography and/or fractional flow reserve > 0.80).	ACh	E Mv	DM in patients with E diffuse vs. E focal vs. Mv vs. without spasm 40 (20%) vs. 4 (10%) vs. 10 (20%) vs. 7 (22%) (*p* = 0.44).
Suda et al. 2019 Japan [77]	187 63.2 ± 12.3 y 113 (60.4%)	Angina-like chest pain; non-obstructive coronary arteries (luminal narrowing < 70% and/or fractional flow reserve > 0.8).	ACh	E Mv	DM in patients with vs. without E spasm 37 (29%) vs. 15 (25%) (*p* = 0.62).
Lee et al. 2017 Korea [78]	4644 55.2 ± 12.5 y 2095 (45.1%)	Typical resting chest pain; no significant coronary artery lesions on angiography (<50% luminal stenosis).	ACh	E Mv	DM in patients with E spasm vs. Mv spasm vs. inconclusive test vs. without spasm 69 (10.7%) vs. 173 (12.6%) vs. 1115 (12.4%) vs. 204 (12.0%) (*p* = 0.644).
Sato et al. 2013 Japan [79]	1760 63.0 ± 11.0 y 903 (51.3%)	Typical or atypical angina-like chest pain.	ACh	E Mv or MvD	DM in patients with vs. without E spasm 179 (21%) vs. 137 (18%) (*p* = 0.183); DM in patients with focal vs. diffuse spasm 110 (22%) vs. 69 (19%) (*p* = 0.383); DM in patients with VA and with vs. without MACE 8 (19%) vs. 171 (21%) (*p* = 0.734).
European studies					
Pellegrini et al. 2022 Netherlands [80]	75 55.17 ± 7.28 y 5 (7%)	Persistent angina in the absence of obstructive CAD.	ACh	E Mv	DM in patients with vs. without spasm 5 (8%) vs. 1 (6%) (*p* = 1).
Seitz et al. 2020 Germany [81]	736 62 ± 12 y 316 (43%)	Stable angina; non-obstructive coronary arteries.	ACh	E Mv	DM in patients with E spasm vs. Mv spasm vs. ACh test inconclusive vs. no spasm 44 (17.8%) vs. 33 (17.8%) vs. 38 (18.0%) vs. 14 (15.1%) (*p* = 0.929).
Montone et al. 2018 Italy [82]	80 63.0 ± 10.7 y 40 (50%)	MINOCA; coronary stenosis < 50% on angiography; suspected coronary vasomotion abnormalities.	ACh or ER	E Mv	DM in patients with vs. without spasm 4 (10.8%) vs. 4 (9.3%) (*p* = 1).
Aziz et al. 2017 Germany [83]	1379 61.87 ± 11.9 y 573 (42%)	Stable angina; non-obstructive coronary arteries.	ACh	E MvD	No association between DM and E spasm and MvD, respectively.
Ong et al. 2014 Germany [84]	921 62 ± 12 y 362 (39.3%)	Diagnostic coronary angiography for suspected myocardial ischemia; no epicardial stenosis ≥ 50%.	ACh	E Mv	DM in patients with vs. without spasm 84 (17.2%) vs. 58 (16%) (*p* = 0.71); DM in patients with E spasm vs. Mv spasm vs. ACh test inconclusive vs. no spasm 50 (18%) vs. 34 (17%) vs. 41 (17%) vs. 17 (15%) (*p* = 0.912).
Ong et al. 2012 Germany UK [65]	124 63 ± 10 y 37 (30%)	Stable angina pectoris; non-obstructive coronary arteries on angiography (0–20% narrowing).	ACh	E Mv	DM in patients with E spasm vs. Mv spasm vs. without spasm 5 (14%) vs. 8 (19%) vs. 12 (26%); (*p* = 0.48).

ACh = acetylcholine; AMI = acute myocardial infarction; CAD = coronary artery disease; DM = diabetes mellitus; E = epicardic; ER = ergonovine; INOCA = ischemia with non-obstructive coronary arteries; MACE = major adverse cardiovascular events; MINOCA = myocardial infarction with non-obstructive coronary arteries; Mv = microvascular; MvD = microvascular dysfunction; NR = not reported; VA = vasospastic angina; ^a^ = without statistical significance.

## Data Availability

Data sharing is not applicable to this article.
